# Emotional temperaments in advanced pregnant goats and its relationship with the feto-maternal blood flow and placentome echotexture

**DOI:** 10.1007/s11259-024-10330-2

**Published:** 2024-02-21

**Authors:** Haney Samir, Ayman A. Swelum, Ahmed Farag, Hossam R. El-Sherbiny

**Affiliations:** 1 Department of Theriogenology, Faculty of Veterinary Medicine, Cairo University, Giza, 12211 Egypt; 2 Department of Theriogenology, Faculty of Veterinary Medicine, Zagazig University, Zagazig, Egypt; 3 Department of Surgery, Anesthesiology, and Radiology, Faculty of Veterinary Medicine, Zagazig University, Zagazig, Egypt

**Keywords:** Calm and nervous goats, Cortisol, Doppler, Xylazine, Maternal and fetal hemodynamics, Placentomes

## Abstract

The current study aimed to investigate the effect of xylazine sedation (non-sedated versus sedated conditions) and animal temperament on the fetal and maternal hemodynamics during the late stage of gestation in goats. In addition, it aimed to study the concentrations of cortisol and the echotexture of the placentome. Fourteen goats were assigned into two equal groups (*n* = 7, each) based on the animal’s emotional temperament (calm versus nervous groups). All goats were examined for assessment of the blood flow within the fetal aorta (FA), umbilical artery (UMA), and middle uterine artery (MUA) using color-pulsed Doppler ultrasonography. Goats were exposed to light sedation using the recommended dose of xylazine (0.05 mg/Kg Bw) intramuscularly. Goats in each group were reassessed for the studied parameters after sedation. Blood samples were drawn to determine the concentrations of cortisol. Placentome echotexture pixel intensity (PXI) was evaluated using computer image analysis software. Results revealed the significant impact of the xylazine sedation on the Doppler indices of the blood flow within the measured arteries (FA, UMA, and MUA), the PXI of placentome echotexture, and cortisol concentrations. The emotional temperament of goats had significant effects on the blood flow parameters of the MUA and UMA, concentrations of cortisol, and the PXI of the placentome. The interaction effect (sedation x temperament) was noticed in the measured parameters of the UMA blood flow, fetal heart rate, and cortisol concentrations. In conclusion, xylazine sedation and emotional temperaments induced alterations in the echotexture of the placentomes as well as the hemodynamic parameters of late-stage pregnant goats without affecting the pregnancy outcomes.

## Introduction

Placental hemodynamics are essential for fetal growth, and they alter according to the stage of gestation and the requirements of fetal development. The last stage of pregnancy is a very critical period because both the placenta and fetus undergo profound maturation processes (Waldvogel and Bleul 2014), and in turn, there are tremendous increases in the placental blood perfusion in a way to adapt to the growing fetal demands (Herzog et al. 2011). Therefore, analysis of changes in the feto-maternal hemodynamics aids in the identification of morphological alterations in the fetoplacental vascular bed (Waldvogel and Bleul 2014).

Invasive techniques like laparotomies and electromagnetic probes were used in earlier studies to examine uterine blood flow (Sakamoto et al. 1996). However, because Doppler ultrasonography allowed scientists and veterinarians to evaluate organ morphology and functions based on vascular perfusion (Lüttgenau and Bollwein 2014; El-Sherbiny et al. 2022; Samir et al. 2023a, b), this technology has seen extensive use in various perspectives of animal reproduction practices. Color Doppler ultrasonography has been enacted for early detection of pregnancy status in buffaloes (Vecchio et al. 2012; Lasheen et al. 2018; Samir and Kandiel 2019; Samir et al. 2019), cows (Pugliesi et al. 2014), and small ruminants (Serin et al. 2010; Elmetwally et al. 2016a). The umbilical artery (UMA) blood flow supplies the fetal section of the placenta with its blood perfusion, while the middle uterine artery (MUA) supplies the majority of the blood supply to the uterus and constitutes the maternal portion of the placental blood perfusion (Herzog et al. 2011).

Under clinical or field circumstances, sedation of nervous animals is generally applied to facilitate the scanning procedures in some cases (Araujo and Ginther 2009). Xylazine is one of the alpha-2 adrenergic agonists that can produce dose-dependent sedation, analgesia, and muscle relaxation (Celestine Okwudili et al. 2014) in numerous veterinary medical practices. Administration of xylazine resulted in decreased stress levels (lower cortisol levels) during the laparoscopic embryo transfer and enhanced the pregnancy rate of recipient goats (Aghamiri et al. 2022). Administration of medetomidine or dexmedetomidine (alpha 2 agonist sedatives like xylazine) in bitches significantly decreased the blood flow to the ovarian arteries and may be a wise alternative to prevent excessive bleeding before the ovariectomy (Nicolás-Barceló et al. 2021). Sedation of animals may be important, especially during the late gestation stage and handling of dystocia cases, to minimize the anxiety fear, and distress, and to facilitate the successful performance of diagnostic or therapeutic procedures, and during conservative or surgical obstetrical procedures, providing patient comfort and cooperation. At the late gestation stage, the accurate blood flow measurement within the MUA, UMA, and fetal aorta (FA) is important to assess the pregnancy status during this critical period, especially in goats due to the high incidence of some metabolic disorders such as pregnancy toxemia. However, it is technically difficult, takes a lot of time, and may need the operator to have certain technical skills as well as the animal’s cooperation (Samir et al. 2021). Indeed, the animal’s temperament and nervous or stressed behavior should also be properly considered. Trembling and fear in some animals might make the examination difficult. Animals that are nervous or stressed out may exhibit tachycardia, which may alter the pattern of the Doppler waveform and affect the accuracy of measurements (Araujo and Ginther 2009). Therefore, sedation of pregnant goats may be needed to fully assess the blood flow within the MUA, UMA, and FA. The purpose of the current study was to ascertain the impact of xylazine, as a sedative agent, on the MUA, UMA, and FA hemodynamic parameters as determined by color-pulsed Doppler ultrasonography. This study’s other objective was to evaluate the impact of temperament (nervous versus calm animals) on the variations in Doppler sonographic measurements of the measured blood vessels before and after xylazine sedation using the recommended dose (0.05 mg/kg body weight; Mandour et al. 2020) in the advanced pregnant goats (last two weeks). Assessment of the echotextural changes of placentomes provides insight into the placental and uterine artery blood flow and placental maturation, particularly in the last days of gestation and approaching the parturition (Can Demi et al. 2022). Therefore, the study also looks into how the xylazine sedation and animal temperament factors affect the echotexture of the placentome and circulating cortisol concentrations.

## Materials and methods

This study was carried out in the Faculty of Veterinary Medicine, Department of Theriogenology, University of Cairo, Egypt. The Ethical Committee for Animal Use and Care at Cairo University’s Faculty of Veterinary Medicine, Egypt, approved all procedures in the current study under the ethical license code: Vet CU 03162023715. In this regard, animals were not exposed to any of the euthanasia procedures during or after the end of the study.

### Animals and management during the study

This research was conducted on Egyptian Balady goats, one of the indigenous breeds of goats in Egypt and numerous adjacent countries. Balady goats are nonseasonal reproductive primarily raised for meat production. Fourteen pluriparous goats (2.65 ± 0.30 years of age; 36.50 ± 1.50 kg of body weight; mean ± SEM) were maintained under natural daylight conditions (the temperature and relative humidity were 20–25 °C and 35–40%, respectively) in a paddock belonging to the Department of Theriogenology, Faculty of Veterinary Medicine, Cairo University. To meet their nutrient needs, goats were given a balanced diet (NRC 2007) consisting of about 900 g of dried alfalfa grass hay and 300 g of concentrates per head per day. The following comprised the basal diet: nitrogen-free extract (47.3%), ash (16.7%), crude protein (13.5%), crude fat (4.4%), neutral detergent fiber (53.2%), and acid detergent fiber (18.06%). In addition, they were allowed to walk outside for two hours per day (morning: 8:00–9:00; afternoon: 16:00–17:00) to relieve the tension of confinement. Every day, the housing bedding was inspected to ensure it remained dry and clean and to replace any that became soiled. There was much room for movement and relaxation in the living arrangements. There were several feeding stations available to guarantee that every goat got the nutrients it needed. Per their breeding records (synchronized estrus, natural breeding, and confirmation of pregnancy assessment by ultrasonography), the goats employed in this study were multiple (twins) pregnant at a late stage (approximately 135 days). Based on a complete clinical examination, goats appeared to be healthy (3-3.5 for BCS), devoid of any gynecological disorders, and disease-free before the start of the study. They were given a prepared ration appropriate for their physiological stage in the form of concentrates and roughages. Water and mineral blocks were freely accessible. Goats were dewormed and vaccinated against the most common endemic infectious diseases by the General Authority for Veterinary Services’ recommendations.

### Experimental schedules

**Fig. 1:**
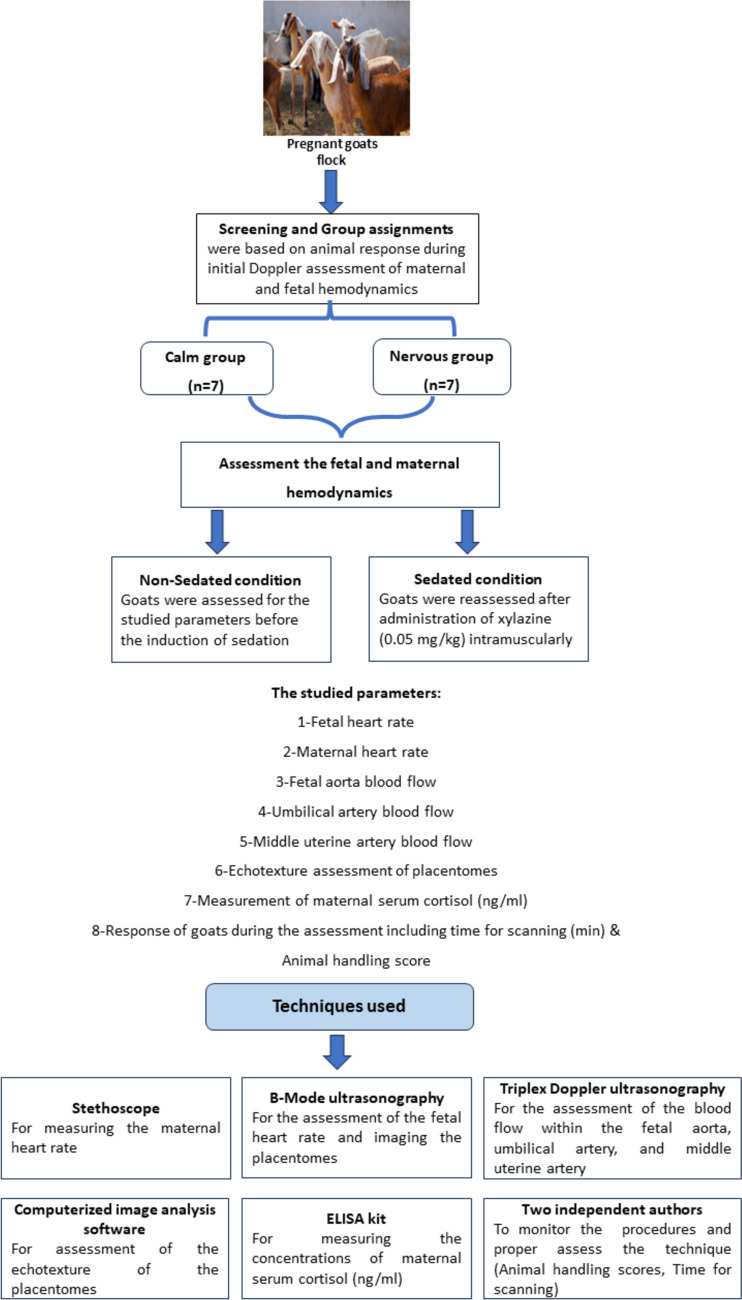
Schematic diagram of the experimental design of this study

Figure 1 represents the schematic illustration of the experimental procedures in this study. In brief, goats under the study were selected from a collection (flock) of late-stage-pregnant goats. After two consecutive initial trials (3–4 days apart) for the assessment of uterine and fetal hemodynamics using color Doppler ultrasonography, goats were categorized based on their behavioral temperament during handling and examination and previous standard criteria (Samir et al. 2023c) into a calm group (*n* = 7) and non-calm or nervous group (*n* = 7). The standard criteria include animal handling scores and time (minutes) required to complete the full assessment of the fetal and maternal hemodynamics by Doppler ultrasonography per goat to reach a proper assessment and grouping of animals. In this regard, the animal handling score is a rating score (from one to five; being five is the most difficult for animal handling during scanning procedures), subjectively assessed by the two independent evaluators, and based on visualizing the response of animals during the scanning procedures in this study including the frequency of animal movement, trials of the animal to escape, frequency of animal cry, and other animal reaction (shivering) during restraining by an assistant for the assessment procedures. After grouping, goats were evaluated for the FA, UMA, and MUA hemodynamics, and echotexture of placentomes before xylazine sedation (non-sedated condition). On a subsequent day, and at about 20 min after sedation with the recommended dosage of xylazine (sedated condition, of 2nd score, Samir et al. 2023c), all parameters were re-evaluated in both groups. In this respect, animals are released following the completion of the study, but they were under our inspection till the due time (parturition) to record any disorders.

### Ultrasonographic assessment of arterial hemodynamics

**Fig. 2:**
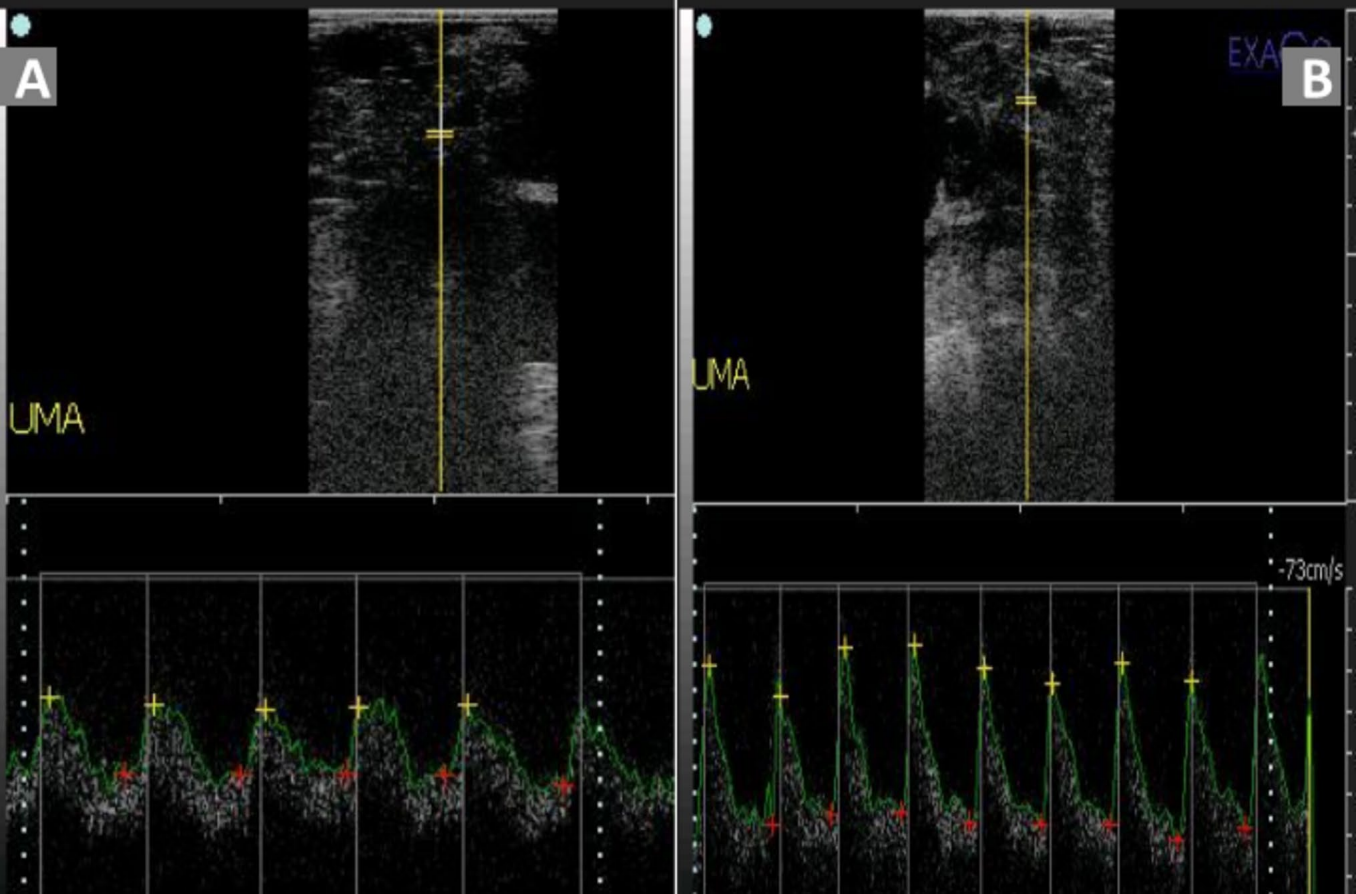
Appearance of the blood flow within the umbilical artery using pulsed Doppler ultrasonography in the late-pregnant goats. Notice the differences between the resistivity impendency between the calm goats (**A**) and the nervous goats (**B**)

**Fig. 3:**
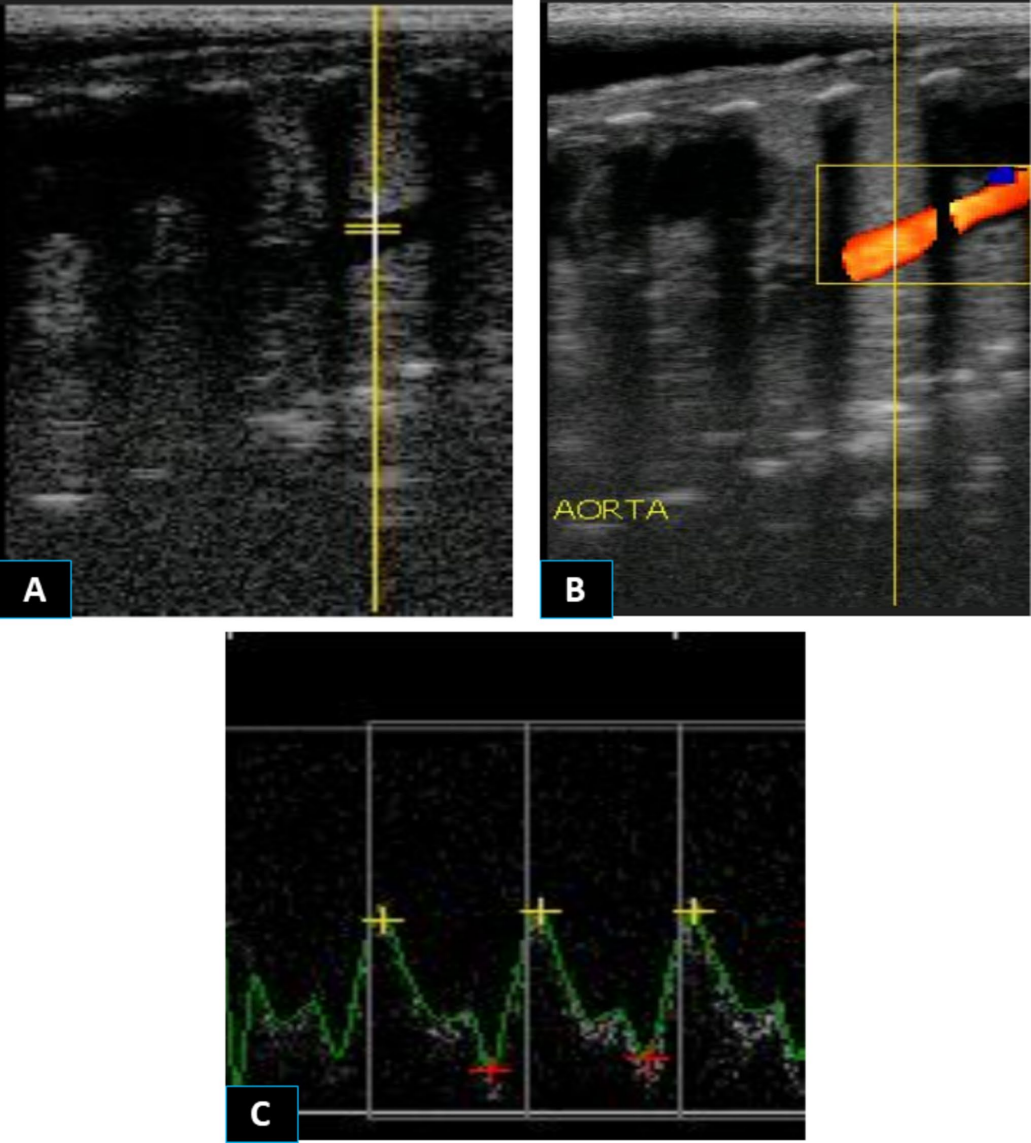
Visualization of the fetal aorta by B-mode (**A**) and color mode (**B**) ultrasonography and the assessment of the waveforms of the blood flow within it by pulsed Doppler ultrasonography (**C**) in late-pregnant goats

All ultrasonographic scanning was performed by the same operator (first author) who has experience in reproductive ultrasonography in farm animals using an ultrasound device (ExaGo, IMV, France) supplemented with color and pulsed Doppler mode and a linear transducer (5-7.5 MHz). To enable better visualization and assessment of the umbilical artery and fetal structures by ultrasonography, goats’ ventral abdominal surfaces (from the area next to the udder to the last rib) were clipped and shaved one day prior to the assessment day to avoid the effect of such stressor on the measured parameters in the first evaluation. Goats were examined after laying laterally (Gonzalez-Bulnes et al. 2010; Ramírez-González et al. 2023) on a comfortable mat table and restrained carefully by an assistant to avoid the compression of the aorta. Examinations were started abdominally to evaluate the hemodynamic changes of the UMA and FA following previous studies (Serin et al. 2010; ElMetwally et al. 2016a). Briefly, the UMA was identified by B-mode ultrasonography based on the recognizable anatomical feature of the umbilical cord (the presence of 4 vessels; 2 arteries, and 2 veins) floating near the abdomen of the fetus (Fig. 2). For evaluating the FA hemodynamics, the sagittal section of the fetal chest was visualized by B-mode ultrasonography and adjusted to locate the FA originating from the heart and crossing longitudinally through the chest of the fetus (Fig. 3). Once the UMA or FA was located and visualized by B-mode ultrasonography, Doppler color flow mode was activated, followed by turning on the pulsed wave mode to monitor the arterial spectral waves with clear systolic and diastolic appearances. The following parameters were measured automatically and recorded: Resistive index (RI), pulsatility index (PI), and systolic/diastolic ratio (S/D). Fetal heart rate (FHR) was measured by ultrasonography following the methods of Karen et al. (2009) and Karadaev et al. (2021). After visualizing the fetal heart with B-mode ultrasonography, the ultrasonic device was switched to B- and M-mode simultaneously to obtain an accurate measurement of the fetal heart rate, which is determined by measuring the distance between two heart waves—either from crest to crest or from trough to trough, depending on which part of the wave was more pronounced. The maternal heart rate (MHR) was determined using a stethoscope.

**Fig. 4:**
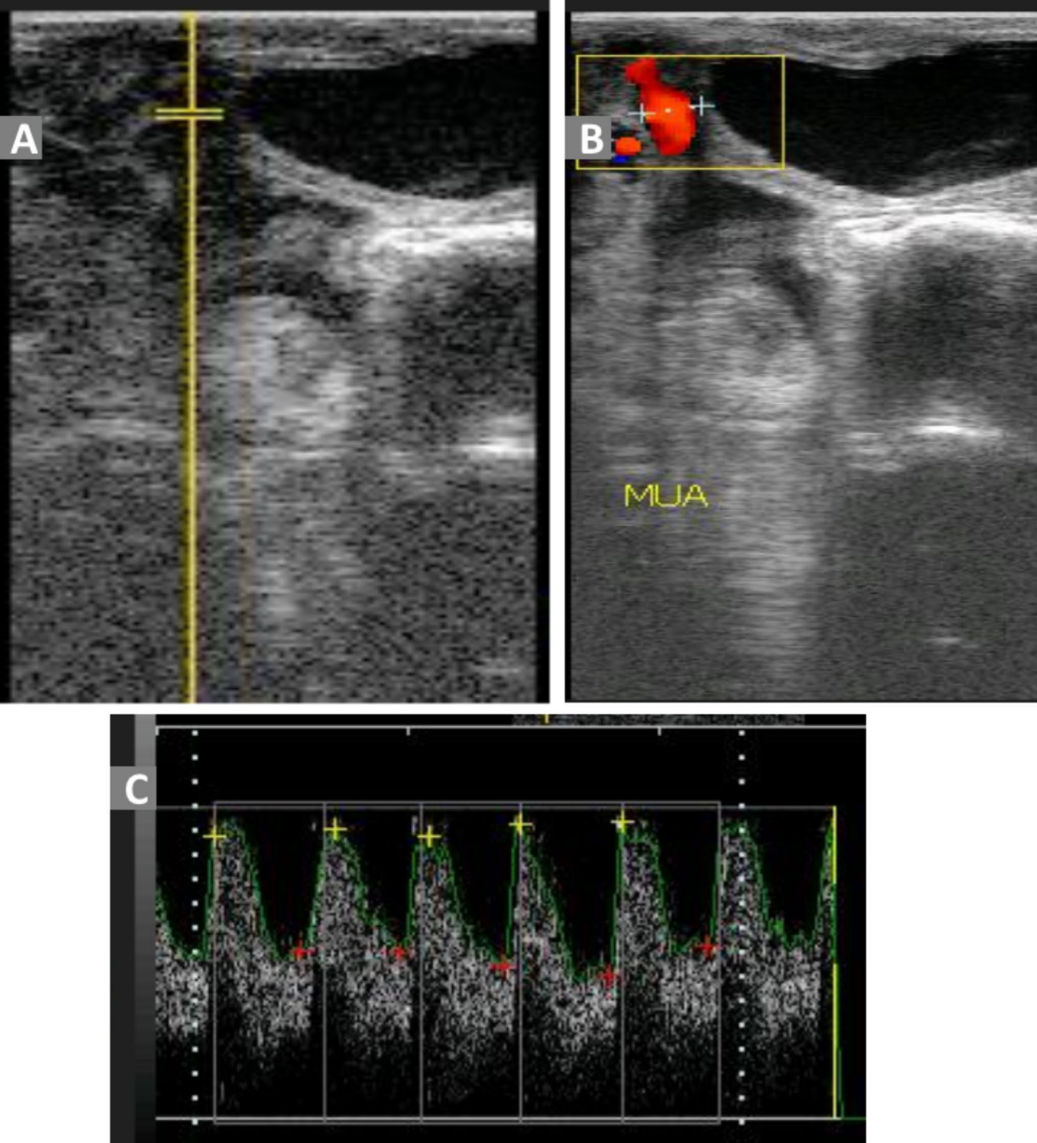
Imaging the middle uterine artery by B-mode ultrasonography (**A**), color Doppler ultrasonography (**B**), and the spectral forms of the blood flow within it by pulsed Doppler ultrasonography (**C**) in late-pregnant goats

For evaluating the blood flow within the MUA, the transrectal approach was adopted using a transrectal linear transducer that had been altered by taping with a wooden rod to regulate the positioning of the ultrasonographic probe inside the rectum. When necessary, the rectum was cleaned of feces before the transducer was introduced and maneuvered into the rectum using a coupling ultrasonic gel. To visualize the MUA by B-mode ultrasonography, the lubricated transducer was spun 90° clockwise and 180° in the opposite direction after being inserted into the rectum. The location of the MUA is craniolateral to the anechoic urinary bladder, close to the external iliac vessels as a reference guide (Fig. 4). The uterine artery was visualized in both directions in a cross or longitudinal section by positioning the transducer laterally and dorsally to the iliac artery branch (Beltrame et al. 2017). The hemodynamic evaluation of MUA was performed as those reported for the UMA and FA. In case of maternal restlessness, movement, cardiac arrhythmia, or frequent fetal movement, the evaluation was discontinued for a while and recontinued later. At least, three minimum measurements were taken for each Doppler parameter for evaluating the blood flow within the MUA, and the mean values were considered. The ultrasound scanner automatically recorded and preserved the ultrasound images and all pertinent measurements. All settings of the ultrasound scanner were standardized and fixed for all scanning procedures, the high pass filter was adjusted at 150 Hz, and the gate of the Doppler angle with the examined vessels was adjusted constantly at 1.5 mm with an insonation angle of less than 60°. Pulse repetition frequency (PRF) was 4000 Hz.

### Computerized assessment of the echotexture analysis of placentomes

**Fig. 5:**
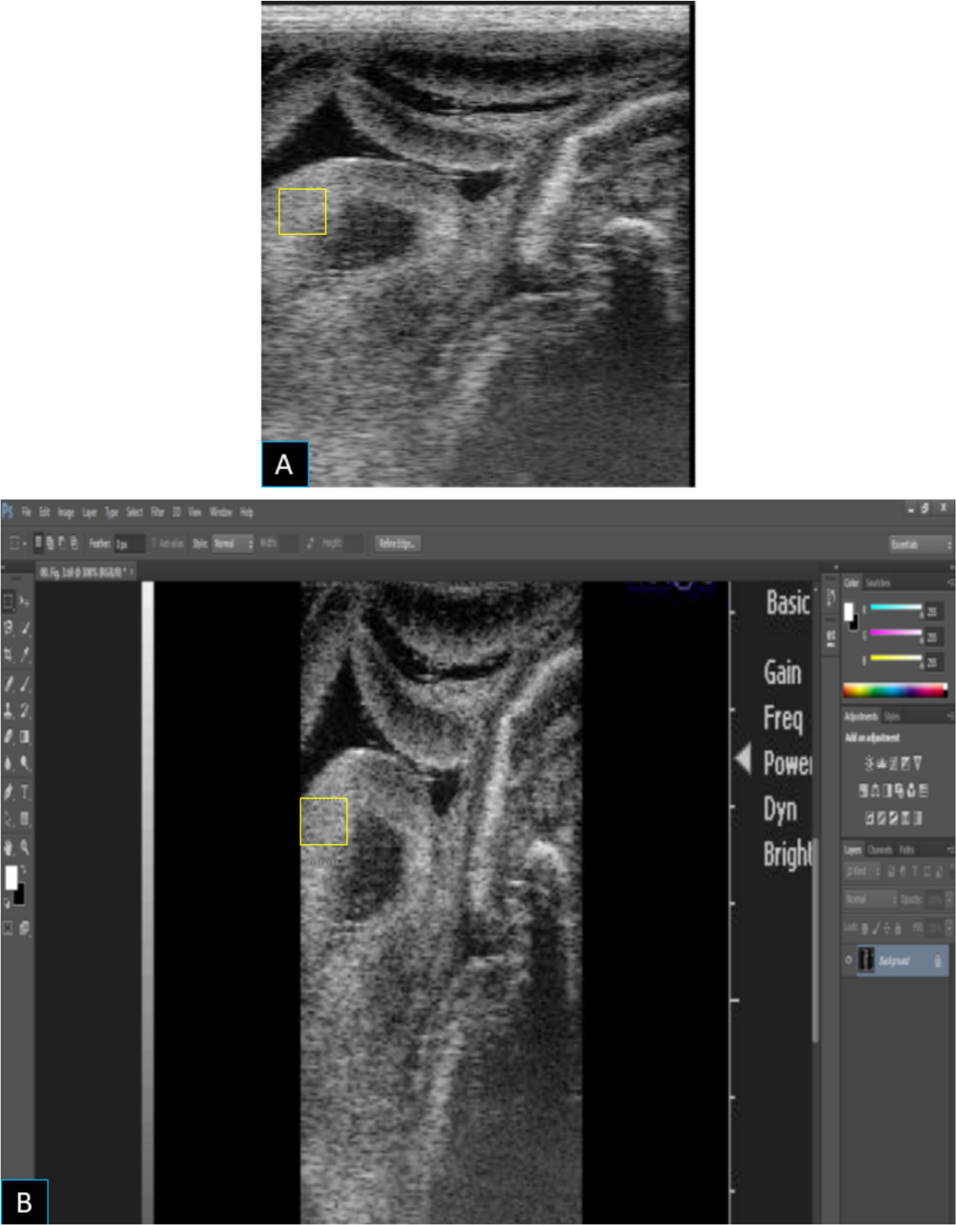
Imaging the placentomes by B-mode ultrasonography (**A**) and the assessment of the echotexture parameters using a computerized image analysis software (**B**)

Good images of B-mode ultrasonography of the placentomes (2–3 ones near the fetus) were snapped frozen and stored for further echotexture evaluation using a computer image analysis software (Adobe Photoshop CC software, USA) as previously stated (Polat et al. 2015; Samir et al. 2023d). Briefly, 3 square spots (0.5 × 0.5 cm) were positioned on the placentome picture while avoiding artifacts (Fig. 5), and the software algorithm calculated the pixel intensity (PXI) and the integrated density (IGD) of the placentome echotexture. The PXI of the placentome corresponds to the average pixel values within the selected area of the placentome tissues and could reflect its blood perfusion based on a reverse digital scale of gray shades ranging from one to 255; the value of one corresponds to black while the number 255 corresponds to white (Samir et al. 2020; Can Demi et al. 2022).

### Assessment of the response of animals during the experimental procedures

The effect of xylazine sedation (non-sedated versus sedated conditions) and the maternal emotion (calm versus nervous groups) on the response of animals during handling for Doppler assessment of the feto-maternal hemodynamics including the animal handling score and the time required to complete the scanning procedures were reported.

### Blood sampling and hormonal analysis

Blood samples (about five ml) were withdrawn from the jugular vein, kept at room temperature for clotting, and then centrifuged at 3200 rpm for 15 min to harvest the sera. The serum samples were then separated, aliquoted, and kept at − 20 °C to determine the concentrations of cortisol (ng/ml) using a commercial ELISA kit (DRG® Cortisol ELISA Kit, EIA1887R) following the guidelines of the manufacturers (BioCheck, Inc. Foster City, USA). The range of measurement is between 1.3 and 800 ng/ml, while the sensitivity of the kit is 2.5 ng/ml.

### Statistical analysis

Data distribution was first assessed using the Shapiro-Wilk test (Mishra et al. 2019). In this study, a two-way analysis of variance (ANOVA) test was used to analyze the effect of temperament, sedation, and their interactions followed by a post hoc test (Bonferroni test). All results were expressed in the form of mean ± standard error. All statistical analyses were performed using GraphPad Prism 5 (San Diego, CA, USA). A value of the probability of less than 0.05 was considered a significant difference.

## Results

First of all, monitoring the animals during the experimental procedures and after the study’s completion until the due time (parturition) documented no incidences of abortion, fetal deaths, stillbirth, or dystocia issues. Table 1 shows the impact of animal temperament, sedation, and their interactions on the FHR and FA blood flow parameters (RI, PI, and S/D). There was a significant impact of the sedation factor (*P*<0.0001) on the studied parameters, while the animal temperament factor did not affect (*P*˃0.05). The FHR was lower, while the examined parameters of the FA blood flow were considerably higher in the sedated conditions than in the non-sedated ones (*P*<0.0001). The interaction between the two factors was observed in the FHR (*P*<0.05). The FHR was higher (*P*<0.05) in the sedated nervous group (133.57 ± 2.58 beats/minute) than in the sedated calm group (115.29 ± 1.29 beats/minute).

**Table 1:** Effect of the sedation (xylazine 0.05 mg/kg B.W) and temperament on the fetal heart rate and fetal aorta (FA) blood flow as assessed by color pulsed Doppler ultrasonography in the late pregnant goats (135 days of gestation)

Effects	Groups	*n*	Fetal heart rate (beat/minute)	FA blood flow-RI	FA blood flow-PI	FA blood flow S/D
Temperament effect	Calm	14	137.50 ± 6.43	0.69 ± 0.04	1.31 ± 0.10	3.92 ± 0.46
	Nervous	14	147.79 ± 4.48	0.70 ± 0.03	1.34 ± 0.37	4.04 ± 0.41
Sedation effect	Before sedation	14	160.86 ± 2.47	0.59 ± 0.02	0.93 ± 0.04	2.49 ± 0.08
	After sedation	14	124.43 ± 2.89*	0.80 ± 0.02*	1.72 ± 0.05*	5.47 ± 0.19*
Interaction effect	Calm before sedation	7	159.71 ± 3.61^a^	0.57 ± 0.02	0.87 ± 0.07	2.37 ± 0.12
	Calm after sedation	7	115.29 ± 1.29^b^	0.82 ± 0.02	1.75 ± 0.09	5.47 ± 0.33
	Nervous before sedation	7	162.00 ± 3.61^a^	0.61 ± 0.02	0.99 ± 0.02	2.61 ± 0.10
	Nervous after sedation	7	133.57 ± 2.58^c^	0.78 ± 0.02	1.68 ± 0.05	5.46 ± 0.21
*P* values	Temperament		0.0810	0.8958	0.6709	0.5855
	Sedation		< 0.0001	< 0.0001	< 0.0001	< 0.0001
	Interaction		0.0117	0.1112	0.1619	0.5583

*RI *Resistive index, *PI *Pulsatility index, *S/D* Systolic/diastolic ratio

Values with * represent the significant effect of sedation (*P* < 0.05)

Values with different superscript letters (a-d) represent the significant effect of interaction between temperament and sedation effects (*P* <0.05)

*n*: The total number of observations/measurements in this study

The sedation, temperament, and interactions had substantial impacts (*P*<0.0001, for all) on the studied parameters of the UMA blood flow (Table 2). In this regard, the RI, PI, and S/D values of UMA blood flow were significantly elevated by sedation (*P*<0.0001). Additionally, the calm group attained lower values of the UMA blood flow parameters compared to the nervous one (*P*<0.0001) either in the sedated or the non-sedated settings. Regarding the interaction factor, the studied parameters of the UMA blood flow were significantly higher in the calm sedated group compared to the calm non-sedated group, while it did not show such effect in the nervous goats (*P*˃0.05).

**Table 2:** Effect of the sedation (xylazine 0.05 mg/kg B.W) and temperament on the umbilical artery (UMA) blood flow as assessed by color pulsed Doppler ultrasonography in the late pregnant goats (135 days of gestation)

Effects	Groups	*n*	UMA blood flow-RI	UMA blood flow-PI	UMA blood flow S/D
Temperament effect	Calm	14	0.44 ± 0.03	0.64 ± 0.06	1.87 ± 0.10
	Nervous	14	0.61 ± 0.01^#^	0.92 ± 0.02^#^	2.58 ± 0.06^#^
Sedation effect	Before sedation	14	0.47 ± 0.04	0.69 ± 0.07	2.03 ± 0.15
	After sedation	14	0.58 ± 0.01*	0.87 ± 0.03*	2.42 ± 0.08*
Interaction effect	Calm before sedation	7	0.34 ± 0.01^a^	0.44 ± 0.02^a^	1.52 ± 0.03^a^
	Calm after sedation	7	0.54 ± 0.01^b^	0.84 ± 0.03^b^	2.21 ± 0.05^b^
	Nervous before sedation	7	0.60 ± 0.01^c^	0.93 ± 0.03^b^	2.54 ± 0.07^c^
	Nervous after sedation	7	0.62 ± 0.01^c^	0.91 ± 0.04^b^	2.63 ± 0.09^c^
*P* values	Temperament		< 0.0001	< 0.0001	< 0.0001
	Sedation		< 0.0001	< 0.0001	< 0.0001
	Interaction		< 0.0001	< 0.0001	0.0001

*RI *Resistive index, *PI *Pulsatility index, *S/D *Systolic/diastolic ratio

Values with # represent the significant effect of temperament (*P* < 0.05)

Values with * represent the significant effect of sedation (*P* < 0.05)

Values with different superscript letters (a-d) represent the significant effect of interaction between temperament and sedation effects (*P* <0.05)

*n*: The total number of observations/measurements in this study

For the MUA blood flow (Table 3), this study revealed a significant effect of the sedation and animal temperament (*P*<0.001 and *P*<0.0001, respectively) on all the studied parameters, while the interaction did not significantly influence (*P*˃0.05). Goats in the sedated condition had higher values of the RI, PI, and S/D of the MUA blood flow compared to the non-sedated (before sedation) one. Regardless of whether goats were sedated or not, the calm goats attained lower values of the RI, PI, and S/D of the MUA blood flow compared to the nervous goats (*P*< 0.0001).

**Table 3:** Effect of the sedation (xylazine 0.05 mg/kg B.W) and temperament on the middle uterine artery (MUA) blood flow as assessed by color pulsed Doppler ultrasonography in the late pregnant goats (135 days of gestation)

Effects	Groups	*n*	MUA blood flow-RI	MUA blood flow-PI	MUA blood flow S/D
Temperament effect	Calm	14	0.48 ± 0.01	0.71 ± 0.02	2.05 ± 0.05
	Nervous	14	0.59 ± 0.01^#^	0.90 ± 0.04^#^	2.44 ± 0.08^#^
Sedation effect	Before sedation	14	0.51 ± 0.02	0.74 ± 0.03	2.08 ± 0.07
	After sedation	14	0.56 ± 0.02*	0.87 ± 0.04*	2.40 ± 0.09*
Interaction effect	Calm before sedation	7	0.44 ± 0.01	0.63 ± 0.02	1.87 ± 0.04
	Calm after sedation	7	0.52 ± 0.01	0.79 ± 0.01	2.22 ± 0.03
	Nervous before sedation	7	0.57 ± 0.01	0.84 ± 0.03	2.30 ± 0.05
	Nervous after sedation	7	0.61 ± 0.02	0.96 ± 0.06	2.58 ± 0.14
*P* values	Temperament		< 0.0001	< 0.0001	< 0.0001
	Sedation		0.0004	0.0005	0.0006
	Interaction		0.2412	0.5976	0.6958

*RI *Resistive index, *PI *Pulsatility index, *S/D *Systolic/diastolic ratio

Values with # represent the significant effect of temperament (*P* < 0.05)

Values with * represent the significant effect of sedation (*P* < 0.05)

Values with different superscript letters (a-d) represent the significant effect of interaction between temperament and sedation effects (*P* <0.05)

*n*: The total number of observations/measurements in this study

The effect of maternal temperament, sedation, and their interactions on the MHR and echotexture of placentomes is presented in Table 4. There were significant effects of animal temperament on the MHR (*P*<0.05), the PXI (*P*<0.0001), and IGD (*P*<0.0001) of placentome echotexture. Regardless of whether subjects were sedated or not, the calm goats had lower heart rates (134.15 ± 6.04 beats/minute) and decreased placentome PXI (81.19 ± 5.37 pixels) and IGD (19149.79 ± 1515.67 pixels) compared to the nervous ones (142.79 ± 5.11 beats/minute, 131.49 ± 5.17 pixel, and 29699.54 ± 2335.19 pixels, respectively). The sedative effect, on the other hand, had significant effects on the MHR and PXI values of the placentome echotexture. Decreased MHR and lower placentome PXI were found in the goats before sedation (156.00 ± 1.99 beats/minute and 97.92 ± 9.95 pixels, respectively) compared to that found after the sedation (120.93 ± 3.76 beats/minute and 114.76 ± 6.56 pixels, respectively).

**Table 4:** Effect of the sedation (xylazine 0.05 mg/kg B.W) and temperament on the maternal heart rate (MHR) and the echotexture of placentomes (pixel intensity: PXI, and integrated density: IND) as assessed by computer analysis software in the late pregnant goats (135 days of gestation)

Effects	Groups	*n*	MHR (beat/minute)	Placentome PXI	Placentome IND
Temperament effect	Calm	14	134.15 ± 6.04	81.19 ± 5.37	19149.79 ± 1515.67
	Nervous	14	142.79 ± 5.11^#^	131.49 ± 5.17^#^	29699.54 ± 2335.19^#^
Sedation effect	Before sedation	14	156.00 ± 1.99	97.92 ± 9.95	25620.50 ± 2311.15
	After sedation	14	120.93 ± 3.76*	114.76 ± 6.56*	23228.83 ± 920.49
Interaction effect	Calm before sedation	7	155 ± 3.30	67.65 ± 5.92	15135.72 ± 1278.51^a^
	Calm after sedation	7	113.29 ± 1.48	94.74 ± 5.37	23163.86 ± 1717.36^b^
	Nervous before sedation	7	157.00 ± 2.45	128.20 ± 9.39	36105.29 ± 3038.31^c^
	Nervous after sedation	7	128.57 ± 6.31	134.79 ± 4.90	23293.80 ± 849.08^b^
*P* values	Temperament		0.0337	< 0.0001	< 0.0001
	Sedation		< 0.0001	0.0181	0.2217
	Interaction		0.0962	0.1353	< 0.0001

*PXI *Pixel intensity, *IND *Integrated density

Values with # represent the significant effect of temperament (*P* < 0.05)

Values with * represent the significant effect of sedation (*P* < 0.05)

Values with different superscript letters (a-d) represent the significant effect of interaction between temperament and sedation effects (*P* <0.05)

*n*: The total number of observations/measurements in this study

**Fig. 6:**
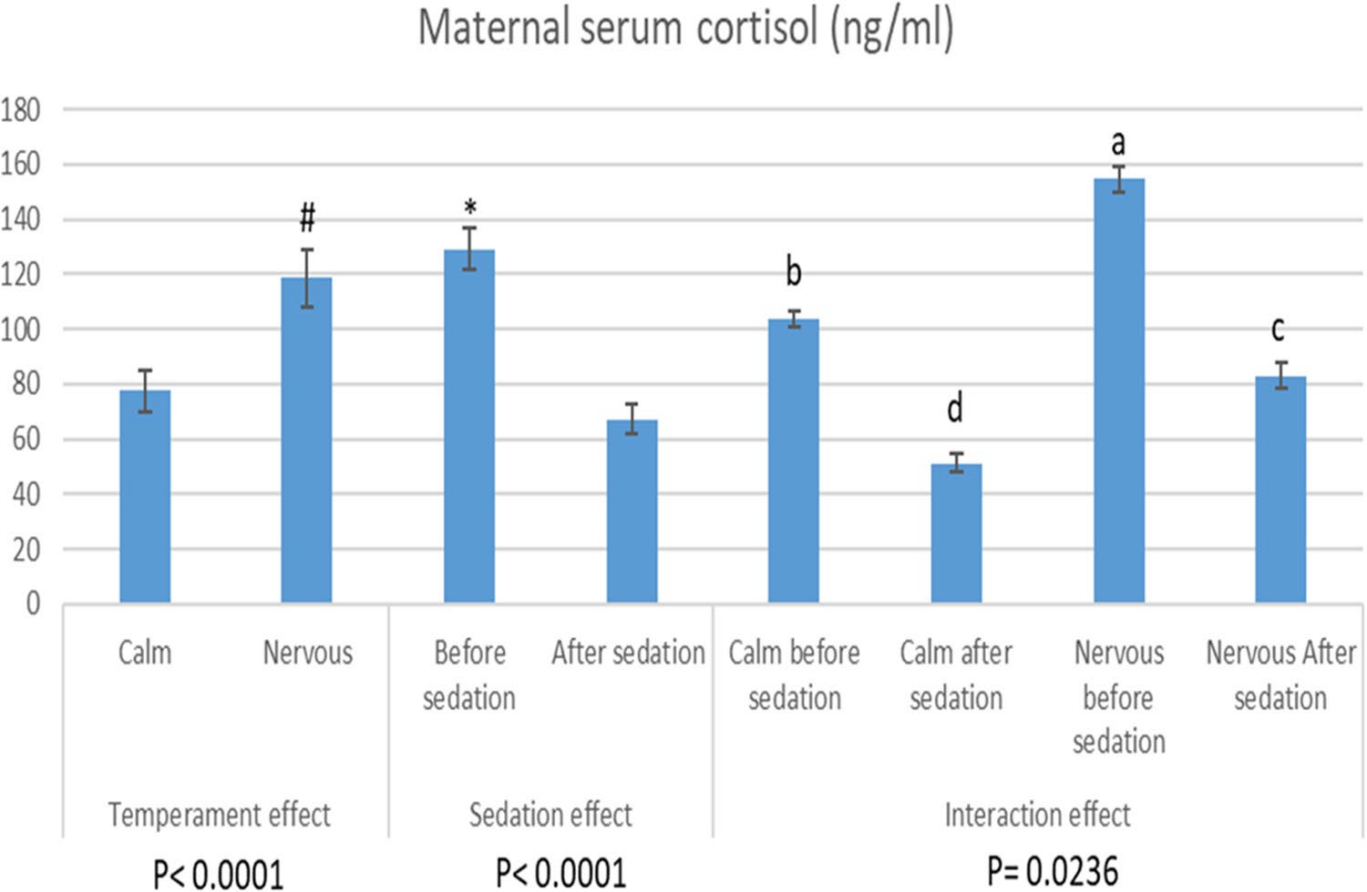
The effect of sedation and the animal temperament on the concentrations of serum cortisol of the late pregnant goats (135 days of gestation). Values with # represent the significant effect of temperament (*P* < 0.05). Values with * represent the significant effect of sedation (*P* < 0.05). Values with different superscript letters (a-d) represent the significant effect of interaction between temperament and sedation effects (*P* <0.05)

Sedation, maternal temperament, and their interactions significantly (*P* < 0.0001, *P* < 0.0001, and *P* < 0.05, respectively) affect the cortisol levels (Fig. 6). Whether under sedation or not, the nervous group’s cortisol levels were considerably greater (118.73 ± 10.43 ng/ml) than those of the calm group (77.51 ± 7.55 ng/ml). Additionally, the sedated goats had lower cortisol concentrations (67.12 ± 5.18 ng/ml) than the non-sedated ones (129.12 ± 7.51 ng/ml). In this regard, calm sedated goats attained the lowest levels of cortisol (51.29 ± 2.97 ng/ml), while the nervous non-sedated goats had the highest levels of cortisol (154.50 ± 4.56 ng/ml), compared to other groups (*P* < 0.05).

Furthermore, temperament, sedation, and their interactions all significantly influenced the time required to complete Doppler scanning (*P* < 0.0001, for all) and the animal handling score (*P* < 0.001, *P* < 0.0001, and *P*  < 0.05) as displayed in Table 5. Both the animal handling score and the time needed to complete the comprehensive assessment of the examined parameters were considerably lower (*P* < 0.05) in the sedated settings (9.38 ± 0.53 min and 1.64 ± 0.13, respectively) compared to the non-sedated ones (21.70 ± 1.95 min and 3.82 ± 0.28, respectively). In the calm group, the time required to complete Doppler scanning, and the animal score for handling were noticeably lower than that of the nervous group (*P* < 0.001). The effect of emotional temperament was noticeable in the non-sedated conditions. Increases (*P* < 0.05) in the time and the animal handling score required to complete the Doppler scanning in the nervous non-sedated goats (27.75 ± 1.84 min and 4.50 ± 0.29, respectively) compared to the calm non-sedated ones (15.65 ± 0.94 min and 3.14 ± 0.32, respectively).

**Table 5:** Effect of the sedation (xylazine 0.05 mg/kg B.W) and temperament on the response of animals during handling for Doppler assessment of the feto-maternal hemodynamics using color Doppler ultrasonography in late-gestation (135 days of gestation) of goats

Effects	Groups	*n*	Time for complete scanning (minutes)	^*^Animal Handling Score
Temperament effect	Calm	14	12.08 ± 1.12	2.21 ± 0.30
	Nervous	14	19.00 ± 2.61^#^	3.25 ± 0.38^#^
Sedation effect	Before sedation	14	21.70 ± 1.95	3.82 ± 0.28
	After sedation	14	9.38 ± 0.53*	1.64 ± 0.13*
Interaction effect	Calm before sedation	7	15.65 ± 0.94^b^	3.14 ± 0.32^b^
	Calm after sedation	7	8.50 ± 0.53^c^	1.29 ± 0.10^c^
	Nervous before sedation	7	27.75 ± 1.84^a^	4.50 ± 0.29^a^
	Nervous after sedation	7	10.25 ± 0.83^bc^	2.00 ± 0.15^bc^
*P* values	Temperament		< 0.0001	0.0002
	Sedation		< 0.00001	< 0.0001
	Interaction		0.0001	0.0184

^∗^Animal Handling Score is a rating score (from 1 to 5), subjectively assessed by two independent evaluators, and based on standard criteria to visualize the response of animals during the scanning procedures in this study. The criteria include the frequency of animal movement, trials of the animal to escape, frequency of animal cries, and other animal reactions (for example, shivering) during restraining by an assistant for the assessment procedures

Values with # represent the significant effect of temperament (*P* < 0.05)

Values with * represent the significant effect of sedation (*P* < 0.05)

Values with different superscript letters (a-d) represent the significant effect of interaction between temperament and sedation effects (at least at *P* <0.05)

*n*: The total number of observations/measurements in this study

## Discussion

Sedatives may be given to animals in clinical circumstances to lessen their distress and make handling them easier for successful diagnostic or therapeutic purposes. Xylazine is widely used as a sedative, analgesic, and muscle relaxant in various species of farm and companion animals. The current study is the first to explore how administering xylazine, a sedative, affects fetal and maternal hemodynamic parameters as determined by color-pulsed Doppler ultrasonography and the echotexture of placentomes during the late stage of gestation in goats. In our study, the sedative goats required less time than the non-sedative ones to perform the comprehensive assessment of fetal and maternal hemodynamics in goats. These findings were consistent with those reported in mares and cows (Araujo and Ginther 2009).

The current study found that xylazine sedation (sedated versus non-sedated conditions) and animal temperament (calm versus nervous animals) had a substantial impact on the examined parameters in late-pregnant goats. In the current investigation. maternal heart rates were substantially lower in the sedated conditions compared to the non-seated conditions. In general, alpha 2-adrenoceptor agonists (such as xylazine) cause a peripheral phase of vasoconstriction, hypertension, and reflex bradycardia, followed by a central phase or effect of decreased sympathetic tone, bradycardia (decreased heart rate), and hypertension (Nicolás-Barceló et al. 2021). A previous investigation on goats found that xylazine sedation significantly reduced heart rate, cardiac output, and Doppler hemodynamic parameters of the pulmonary artery, aortic, and mitral inputs (Mandour et al. 2020). Sedation with xylazine in our study resulted in considerable decreases in blood flow within the MUA, UMA, and FA (as shown by increases in Doppler indices of the blood flow within the examined arteries, such as RI). Doppler indices refer to the impedance of blood flow (Dickey 1997; Blanco et al. 2008). Because of the negative associations between Doppler indices (RI and PI) and the downstream organ blood flow (Dickey 1997; Blanco et al. 2008), increased Doppler indices values suggested lower blood perfusion. Research studies on cows (Hodgson et al. 2002; Waldvogel and Bleul 2014) revealed the negative effects of xylazine sedation on fetal-maternal hemodynamics in the final stages of pregnancy or during parturition. Administration of xylazine significantly reduced arterial blood flow and oxygen pressure in the uterus, increased the RI of the MUA (by 156%) without increasing blood pressure, reduced the blood flow volume of the MUA on both sides (by about 6–11%). In addition, it induced reductions in the blood flow volume and the pulse rate of the maternal (17%) and fetal (6%) sides of the umbilical arteries. The decreased heart rate, combined with the lowered blood pressure, and the aforementioned effects may result in hypoxia in peripheral organs (i.e. the uterus) and lowered oxygen intake by the fetus by about 59% (that may be due to a decrease in hematocrit, too), and finally leads to a decrease in blood pumping (Hodgson et al. 2002), and transient fetal hypoxia and probability of death (Waldvogel and Bleul 2014).

The decreased blood flow within the MUA that was found in the present study is consistent with previous reports in cows (Waldvogel and Bleul 2014), mares (Gibbs and Troedsson 1995), and guinea pigs (Weiner et al. 1986) after xylazine administration. In contrast to the central effect of xylazine on the cardiovascular system, other studies (Sakamoto et al. 1996; Taneike et al. 1999; Yasuda et al. 2014; Zeiler 2015; Piccinno et al. 2016) referred to the local stimulatory effect of xylazine on the uterine contraction, as a cause for the decreased uterine blood perfusion. It has been reported an increase in intrauterine pressure and uterine contractility following the administration of xylazine in late-pregnant cows (LeBlanc et al. 1984). In goats, considerable decreases in the uterine blood flow, for about one hour, concurrently with increases in the intrauterine pressure were found after xylazine sedation (Sakamoto et al. 1996). In mares, xylazine treatment increases the uterine myoelectric activity (Gibbs and Troedsson 1995), enhances the number and strength of uterine contractions (Zeiler 2015), and could induce a tetanic increase in intrauterine pressure when administered during estrus (De Lille et al. 2000). Using an in vitro tissue bath model of the bovine uterus, researchers found that xylazine increased the intensity of contractions in almost all stages of pregnancy (Piccinno et al. 2016) in a dose-dependent manner (Ko et al. 1990a, b). Interestingly, some studies proposed the oxytocin-like effect of xylazine on the uterus of ruminants (Rodriguez-Martinez et al. 1987; Marnet et al. 1987; Pirnik et al. 2005). Xylazine administration has the same effect on increasing intrauterine pressure as oxytocin (Pirnik et al. 2005) in non-pregnant cows and the greatest effects occurred in the proestrus phase (Rodriguez-Martinez et al. 1987). Xylazine-induced uterine contraction in cows is directly mediated by α-2 adrenoreceptors in the myometrium (Ko et al. 1990b). This action is achieved through selective activation of postsynaptic-2 adrenergic receptors in the uterus, as well as numerous neurotransmitters such as catecholamines and acetylcholine (Sakamoto et al. 1996; Zeiler 2015). When these neurotransmitters bind to alpha-adrenergic and muscarinic receptors, they increase uterine contraction (Yasuda et al. 2014; Taneike et al. 1999; Piccinno et al. 2016). Smooth muscle contraction caused by activation of these receptors is mediated by adenylate cyclase inhibition. As a result, less cAMP is produced, which increases the cytoplasmic calcium availability by opening calcium channels (Ko et al. 1990a, b; Perez et al. 1997).

Collectively, xylazine may have a local effect on the uterus through increased uterine contraction and intrauterine pressure, or a central effect through its detrimental effects on the cardiovascular system (decreased heart rate, cardiac outputs, and other negative effects on the vascular system). Because of this, xylazine sedation during the final stages of pregnancy or labor for the diagnostic purposes of surgical interventions (such as a cesarean section) might result in devastating fetal hypoxia and the possibility of abortion, premature birth or neonatal mortality (Hodgson et al. 2002; Piccinno et al. 2016). Even in rats, intraperitoneal treatment of xylazine (10 mg/kg BW) for three consecutive days of gestation (19–21 days) caused 18–25% of fetal losses, which may be related to fetus hypoxia and increasing uterine contractions (Zohani-Ghayeni et al. 2023). However, in the current study, all goats delivered normally without recording any dystocia cases or fetal losses, or abortion. Similar findings were found in an old study (Sakamoto et al. 1996) using four times the dosage of xylazine we used in our study, although it greatly decreased the MUA blood flow. They argued the existence of a compensatory mechanism at the placental level, which protects the fetus from the undesirable effects of xylazine (Sakamoto et al. 1996; Jansen et al. 1984). The usage of xylazine sedation in the non-pregnant animal may not be as serious as in the pregnancy. Sedation with detomidine (a more potent and rather specific alpha 2-adrenoceptor agonist in the central and peripheral nervous systems than xylazine) in pony mares and xylazine in Holstein heifers did not induce variations in the local vascular perfusion of the ovaries and endometrium (Araujo and Ginther 2009), despite they reduced heart rate and the blood flow volume and velocity within the internal iliac artery.

Regarding the animal temperament, the Doppler indices of blood flow within the MUA and UMA were lower in the calm group compared to the nervous group. These results indicate higher blood perfusion within the MUA and UMA in the calm goats than in the nervous goats. Our findings matched those of other investigations (Aberdeen et al. 2010; Vythilingum et al. 2010; Elmetwally et al. 2021). It was reported the impact of maternal anxiety on the blood flow within the MUA and UMA in sheep and goats, especially during the second half of gestation (Elmetwally et al. 2016b). In our investigation, there were no discernible differences in the fetal aortic blood flow or FHR between the calm and nervous groups. Elmetwally et al. (2021) did not find a significant impact of maternal anxiety on the FHR during pregnancy. Similar findings were observed in humans during late pregnancy (DiPietro et al. 2010; Lobmaier et al. 2020). On the opposite, others reported increased FHR in pregnant women of high trait anxiety (Monk et al. 2000; Sjöström et al. 2002; Makino et al. 2009). Indeed, the association between the FHR and maternal emotion is situational (Ask et al. 2019; DiPietro et al. 2007), and there may not be a rigid relationship between the FHR and MHR (DiPietro et al. 2007). In general, the relationship between maternal psychology and normal pregnancy physiology is complex and may be influenced by a variety of circumstances (Mendelson et al. 2011).

In the present study, the interaction effect of sedation and animal temperament revealed noticeable decreases in the Doppler indices of the UMA blood flow in the calm sedated group compared to the calm non-sedated one, while the nervous group did not present such an effect. The authors thought the xylazine dose that was used in the present study may be enough only to relieve the animal distress in the nervous group without inducing significant changes in the hemodynamic parameters of the UMA. Indeed, the nervous goats attained high levels of cortisol either in the sedated or the non-sedated conditions compared to that in the calm one. Similar findings were reported in the pregnant sheep and goats (Elmetwally et al. 2016b, 2021). Previous studies in cows (Herzog et al. 2011), small ruminants (Elmetwally et al. 2016b, 2021), and humans (Fan et al. 2018) found a close link between increased animal anxiety, cortisol levels, and decreased uterine artery blood flow. Xylazine sedation significantly reduced the maternal serum cortisol in both the calm and nervous goats. Similar findings were found in dogs (Väisänen et al. 2002), and goats (Aghamiri et al. 2022; Samir et al. 2023c) after sedation with xylazine. Decreased cortisol levels may be attributed to the depressive effect of sedation on the central nervous system and the adrenocortical stimulation caused by emotional stress (Oyama 1973; Sanhouri et al. 1992; Tranquilli et al. 2007). Since elevated cortisol levels have been shown to increase heart rate and cardiac output, several studies reported a direct relationship between cortisol levels and cardiovascular function (Kelly et al. 1998; Whitworth et al. 2005; Morris et al. 2016).

This study assessed the effects of sedation and animal temperament on the echotextural of placentomes (PXI), as a good indicator of placental blood perfusion and function (Can Demi et al. 2022). The lower PXI of the placentome echotexture in the present study’s calm group compared to that in the nervous group may be due to the placentomes’ greater blood perfusion as a result of the higher MUA blood flow. Increased blood perfusion may increase the vascular permeability of placentomes, reflecting their high-water content, and eventually leading to declines in the PXI values of placentome echotexture. Our explanation is pursuant to those argued in previous works of literature related to testicular echotexture in small ruminants (Ungerfeld and Fila 2011; Samir et al. 2020). Because of the sensitivity of goats to xylazine (Wagner et al. 1991; Flecknell et al. 2015; Mandour et al. 2020), xylazine sedation might result in rumen atony with bloat and impaired cardiac activity (decreased heart rate, reduced cardiac output, reduced right ventricular contractility, and variation of blood pressure and oxygen tension).

Collectively, xylazine sedation improved the scanning procedures of the feto-maternal blood flow of the late-pregnant goats. However, it induced noticeable changes in the echotexture of the placentomes as well as the hemodynamic parameters of the fetal and maternal sides. Furthermore, maternal anxiety or temperament has significant effects on the studied parameters. Although the present study did not reveal any negative effects of xylazine sedation on the pregnancy outcome in the studied goats, the authors cannot guarantee the absolute safety of xylazine administration on the overall pregnancy status if applied on a large scale of goats. However, xylazine injection could be recommended to induce a temporal sedative effect to facilitate the assessment of uteroplacental hemodynamics in the late stage of pregnant goats in certain cases (accident, nervous or anxious goats, etc.), and following the recommended dose. Evaluating the blood flow within the MUA could be of high priority. However, the blood flow within the FA, UMA, and MUA should be examined to properly assess the uteroplacental and fetal hemodynamics in late pregnant goats. The findings of the present study did not support whether xylazine sedation could be extrapolated to the moment of the delivery or a cesarean section. However, based on previous reports in cows, and due to the high sensitivity of ruminants, xylazine should be administered during the last stages of pregnancy or parturition after exercising extreme caution (Waldvogel and Bleul 2014). Furthermore, it is never recommended to use xylazine for any cases of compromised fetus (prolonged dystocia, placental separation, umbilical cord compression) during obstetrical interferences or birth help.

## Conclusions

In conclusion, xylazine sedation and emotional temperaments induced alterations in the echotexture of the placentomes as well as the hemodynamic parameters of late-stage pregnant goats without affecting the pregnancy outcomes.

## Data Availability

No datasets were generated or analysed during the current study.
